# ER-Phagy and Its Role in ER Homeostasis in Plants

**DOI:** 10.3390/plants9121771

**Published:** 2020-12-14

**Authors:** Yan Bao, Diane C. Bassham

**Affiliations:** 1 Department of Genetics, Development and Cell Biology, Iowa State University, Ames, IA 50011, USA; 2 Department of Biochemistry and Molecular Biology, Michigan State University, East Lansing, MI 48824, USA

**Keywords:** autophagy, endoplasmic reticulum, ER stress, ER-phagy, unfolded protein response

## Abstract

The endoplasmic reticulum (ER) is the largest continuous membrane-bound cellular organelle and plays a central role in the biosynthesis of lipids and proteins and their distribution to other organelles. Autophagy is a conserved process that is required for recycling unwanted cellular components. Recent studies have implicated the ER as a membrane source for the formation of autophagosomes, vesicles that transport material to the vacuole during autophagy. When unfolded proteins accumulate in the ER and/or the ER lipid bilayer is disrupted, a condition known as ER stress results. During ER stress, ER membranes can also be engulfed through autophagy in a process termed ER-phagy. An interplay between ER stress responses and autophagy thus maintains the functions of the ER to allow cellular survival. In this review, we discuss recent progress in understanding ER-phagy in plants, including identification of regulatory factors and selective autophagy receptors. We also identify key unanswered questions in plant ER-phagy for future study.

## 1. Introduction

Plants live in a world of ever-changing conditions; for survival, they need to adapt to the challenges of their surroundings to balance growth and stress responses [1,2]. At the cellular level, this balance requires the cooperation of multiple organelles. The endoplasmic reticulum (ER) is the largest of the endomembrane organelles, and makes connections with almost all other organelles [3]. It provides docking sites for ribosomes, is a major site of protein and lipid synthesis, and therefore generates membrane components that are incorporated into the other endomembrane organelles. Given the critical role of the ER throughout their life cycle, plants, along with other organisms, have developed sophisticated strategies to maintain the homeostasis of the ER, including the unfolded protein response (UPR), ER-associated degradation (ERAD), ER quality control (ERQC) and selective autophagy of the ER, termed ER-phagy or reticulophagy [4,5,6,7]. The majority of research to date has been done using the model plant *Arabidopsis thaliana*, but information on other species is now becoming available [8,9].

## 2. Autophagy and the ER

Autophagy is a pathway that is important for plant stress tolerance, pathogen defense, and nutrient remobilization during senescence. It is intimately associated with the ER, which both provides membrane for autophagosome formation and is itself degraded by autophagy during ER stress or nutrient starvation conditions. Upon activation of autophagy, double-membrane structures called autophagosomes form at the ER and envelop unwanted or damaged cellular components, including organelles, transporting them to lysosomes or vacuoles for degradation or recycling [10]. Extensive progress has been made in deciphering the mechanism of autophagy since the identification of the autophagy machinery via yeast genetic screens, and most of the key autophagy components are found in multicellular organisms, including plants and animals [11]. Formation of autophagosomes requires the collaboration of a series of autophagy-related (ATG) proteins and associated complexes: (1) the ATG1-ATG13 complex senses the signals and initiates autophagosome formation; (2) ATG9 and associated proteins acquire lipids for the expansion of the phagophore, a cup-shaped double-membrane sequestering compartment that expands to form an autophagosome; (3) the phosphoinositide 3-kinase (PI3K) complex generates phosphatidylinositol 3-phosphate (PI3P) for autophagosome nucleation; (4) the ATG5-ATG12-ATG16 complex acts as an E3 ligase in the conjugation of ATG8 and phosphatidylethanolamine (PE); and (5) ATG8-PE participates in membrane expansion and cargo selection [12]. ATG8 is a key adaptor that can recognize selective autophagy receptors or cargo through either its ATG8-interacting motif (AIM) [13], or a second recently-identified distinct ubiquitin-interacting motif (UIM) [14].

## 3. The ER as an Autophagosome Membrane Source

Determining the membrane source for autophagosome formation is a key in understanding the mechanism underlying autophagy initiation in plants. Accumulating evidence suggests that autophagosomes form from the ER in *Arabidopsis* and are physically attached to the ER membrane at early stages. A number of factors have been identified as working in autophagosome formation or expansion at the ER, although how they act together to enable this process is in most cases unclear. Because of the dual role of the ER as membrane source and cargo for autophagosomes, care must be taken in distinguishing between these possibilities when studying the relationship between the ER and autophagy.

*Arabidopsis* ATG5 is an essential autophagy component and is present at the phagophore from initiation. During autophagy induction, a ring of ATG5 defines the edge of the growing phagophore, narrowing as the autophagosome closes. The movement and expansion of the phagophore is tightly and asymmetrically connected to that of the cortical ER underneath, suggesting a close physical relationship between nascent autophagosomes and the ER [15].

Out of the autophagy core machinery, ATG9 is the only integral membrane protein, and is therefore a good candidate for a role in membrane delivery and expansion. Structural analysis of *Arabidopsis* ATG9 indicates that it can form a homotrimer, with monomers interacting via their C-terminal, membrane-embedded cytoplasmic regions [16], and in yeast and humans, ATG9 has been shown recently to act as a lipid scramblase [17]. In an *Arabidopsis atg9* mutant under conditions of autophagy induction, autophagosome-related tubular structures with direct membrane continuity with the ER are dramatically extended and accumulated, again suggesting a continuity between forming autophagosomes and the ER. Trafficking of the phagophore-associated autophagy protein ATG18a is greatly compromised, suggesting a dysfunction in normal autophagy progression in this mutant. Meanwhile, a transient membrane association between ATG9-containing vesicles and the autophagosome can be seen. ATG9 thus plays an essential role in ER-derived autophagosome formation in plants [16,18].

SH3P2 (SH3 DOMAIN-CONTAINING PROTEIN2) belongs to the Bin-Amphiphysin-Rvs (BAR) domain-containing protein family, and associates with membranes by binding to PI3P. During autophagy induction, SH3P2 is recruited to ER-derived cup-shaped structures, where it is involved in deforming the lipid bilayer to generate autophagosomes. Moreover, SH3P2 associates with the PI3K complex and promotes formation of foci containing PI3K, thereby facilitating autophagosome membrane expansion or maturation. Repression of SH3P2 expression suppresses autophagy, indicating that it has a key role in ER-associated autophagosome biogenesis [19].

A role for the actin cytoskeleton in autophagosome formation at the ER has been proposed. The *Arabidopsis* EH (Epsin15-homology) proteins are components of the TPLATE complex (TPC) and localize to both the plasma membrane and autophagosomes. During autophagy induction, actin, clathrin, AP-2 subunits, ARP2/3 complex and TPC can be recruited to autophagosomes by the EH proteins. Interestingly, these proteins interact with VAP27-1, a vesicle-associated membrane protein-associated protein, at ER-PM contact sites (EPCS) to regulate autophagosome formation, indicating a potential for EPCS as the membrane initiation site for autophagosome formation. Both EH1- and VAP27-1- deficient mutant plants are more susceptible to nutrient deficiency, consistent with defective autophagy [20]. The SCAR/WAVE complex component NAP1 also links the ER, actin and autophagy. It localizes to vesicles that are both ER- and cytoskeleton-associated, and upon autophagy induction, co-localizes with ATG8, suggesting a role in recruiting membrane for autophagosome formation. Indeed, fewer autophagosomes were observed in a *nap1* mutant during nutrient starvation, and the mutant was more sensitive to stress, similar to autophagy mutants [21]. Together, these studies suggest that the actin cytoskeleton is actively involved in autophagosome formation via membrane recruitment at the ER.

## 4. Function of Plant ER Stress Response Pathways in ER Homeostasis 

De novo biosynthesis of proteins, and their subsequent correct folding and targeting, are crucial for their functions to support the plant life cycle. However, various environmental stresses, for example heat and pathogen infection [22], can cause protein misfolding or unfolding in the ER. For survival, plant cells have evolved multiple ERQC systems to handle these challenges [23]. One such system is ERAD, a pathway that can recognize misfolded proteins directly and target them for proteasomal degradation [24]. When the ER is loaded beyond the capacity of the protein folding machinery, ER stress is evoked, which triggers an unfolded protein response (UPR) [25]. In plants, two arms of the UPR signaling pathway work cooperatively, including (1) cleavage of the mRNA encoding bZIP60 by IRE1, through its ribonuclease domain, to generate a nuclear-targeted transcription factor and (2) S1P- and S2P-mediated proteolytic cleavage of membrane-anchored bZIP17 and bZIP28 to again generate nuclear-targeted transcription factors. These three bZIP transcription factors can homo- and/or heterodimerize to work cooperatively, inducing downstream expression of genes such as those encoding molecular chaperones that increase the folding capacity in the ER [4]. In addition to the two shared UPR pathways mentioned above, mammals have another distinct UPR signaling pathway mediated by protein kinase RNA-like ER kinase (PERK), which does not appear to be present in plants [26]. Disruption of UPR sensing and signaling leads to defects in plant growth and development, as well as an inability to respond to stressful conditions [27,28,29].

Upon severe ER stress, dysfunctional ER membranes are degraded by a type of selective autophagy termed ER-phagy [30], which acts cooperatively with the UPR to degrade unfolded proteins [31]. ER-phagy, together with the ERAD and UPR pathways, contributes to the maintenance of ER homeostasis, which when disrupted may threaten plant growth, development or stress responses.

## 5. Selective Autophagy of the ER

The last few years have seen substantial progress in our understanding of ER-phagy in plants [5]. *Arabidopsis* ER-phagy can be demonstrated by the treatment of *Arabidopsis* seedlings with the ER stress agents tunicamycin (TM) or dithiothreitol (DTT). Under these conditions, the ER undergoes morphological changes in structure, and fragments of the ER are delivered to the vacuole by autophagosomes [30]. ER-phagy is also triggered by heat stress and by expressing an artificial unfolded protein mimic in the ER, both of which cause ER stress [31]. Overexpression of molecular chaperones or addition of chemical chaperones can block this autophagy induction, strongly suggesting that the accumulated misfolded or unfolded proteins in the ER are the signal for ER-phagy [31]. The ER stress sensor Inositol-Requiring Enzyme-1b (IRE1b) is essential for ER stress-induced ER-phagy, while bZIP60, the classic target of IRE1b, is not required [30]. IRE1b contains both RNase and protein kinase domains, and the RNase activity, but not the protein kinase activity, of IRE1b is critical for ER stress-induced autophagy. Transcriptome analysis of an *ire1b* mutant identified several regulated IRE1-dependent decay (RIDD) targets that potentially repress autophagy during normal conditions, while during ER stress, they are degraded to release this repression [32].

Golgi anti-apoptotic proteins GAAP1 and GAAP3 are anti-programmed cell death proteins that function in the recovery from ER stress by negatively regulating IRE1 activity [33]. Via yeast-two-hybrid screening, membrane-associated progesterone receptor 3 (MAPR3) was identified as a direct interactor of GAAP1/GAAP3. Interestingly, GAAP1/GAAP3 and MAPR3 can physically interact with IRE1b to modulate its activity, thus affecting RIDD and autophagy downstream [34]. A large-scale transcriptome study in maize suggests that during persistent ER stress, a transition from cell survival programs to cell death occurs, with the induction of autophagy occurring in both phases. Autophagy may therefore function both in adaptive responses to ER stress and in programmed cell death upon long-term stress [9]; how this switch from pro-survival to pro-death functions occurs warrants further investigation.

## 6. ER-Phagy Receptors in Plants and Their Functions

Yeast Atg39 and Atg40 are Atg8-interacting ER-phagy receptors that can recognize different ER subdomains for degradation [35]. In mammals, ER-phagy receptors are specified and functionally diverged based on their role in targeting ER subdomains and in stress responses. For instance, FAM134B, RTN3L, ATL3, TEX264 and CALCOCO1 are mainly involved in starvation-mediated ER-phagy, while CCPG1, Sec62 and Epr1 are key players in ER stress-induced ER-phagy [36,37,38,39,40,41,42,43,44,45]. ER-phagy receptors are thus specific to different organisms, and plants appear to lack homologs of some of these receptors. Several ER-phagy receptors have been recently identified in plants, and function in degradation of ER or ER components in response to different conditions. 

**Figure 1:**
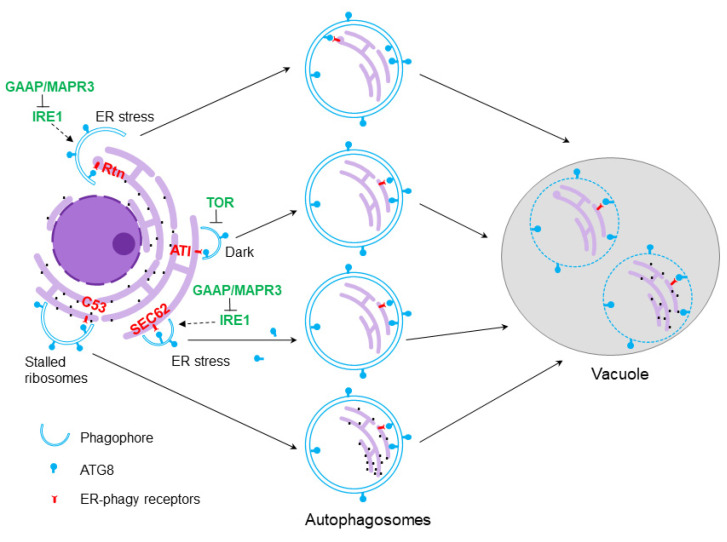
Working model for plant endoplasmic reticulum (ER)-phagy. Multiple receptors function in selecting ER for autophagic degradation: (1) reticulon (Rtn) proteins can remodel ER structure and select ER for degradation during ER stress via binding to highly curved membranes; (2) ATG8-Interacting (ATI) proteins function in dark stress-mediated ER-phagy; (3) SEC62 acts as a receptor for ER-phagy during ER stress; (4) C53-mediated ER-phagy is triggered by stalled ribosomes on the ER. Black dots on the ER denote ribosomes. Potential regulators are shown in green.

SEC62 is an ER-phagy receptor shared by plants and animals. *Arabidopsis* SEC62 is an ER transmembrane protein, disruption of which leads to disorders in plant growth, development and fertility [46]. An *atsec62* null mutant is hypersensitive to ER stress, while overexpression of AtSEC62 confers ER stress tolerance. Importantly, AtSEC62 co-localizes with ATG8 in autophagosomes. The authors thus conclude that AtSEC62 might be an ER-phagy receptor in plants (Figure 1).

The recent discovery of the Arabidopsis cytosolic C53 protein as an ER-phagy receptor provides another example that is shared by plants and mammals. C53 harbors a unique shuffled AIM for interacting with ATG8, and is recruited to autophagosomes upon ER stress. C53 senses the presence of stalled ribosomes at the ER, forming a tripartite receptor complex with the ufmylation ligase components UFL and DDRGK1, resulting in the degradation of the nascent ER proteins and relieving the stress. The corresponding mutants are therefore more ER stress-sensitive. C53 thus provides an alternative quality control pathway for maintenance of ER homeostasis [47].

Reticulons (Rtns) are a family of conserved eukaryote-specific proteins residing predominantly in the ER. A critical role for Rtn proteins in ER turnover and autophagy has been discovered in mammals and yeast, and recently shown also in maize endosperm aleurone cells [8]. Previously, *Arabidopsis* RTNLB13 had been demonstrated to be involved in remodeling ER architecture [48], and a recent study of endosperm-expressed and ER-localized maize Rtn1 and Rtn2 suggests a function in common in this regard. Interestingly, four AIM motifs (one in the C-terminus, one in the cytoplasmic loop, and two within the transmembrane segments) were found in maize Rtn proteins, which mediate interaction with ATG8 proteins. The binding of Rtn2 to ATG8 is increased upon ER stress, suggesting a potential for maize Rtn1 and Rtn2 to act as ER-phagy receptors [8].

The plant-specific trans-membrane ATG8-Interacting (ATI) proteins ATI1 and ATI2 function in the removal of damaged chloroplasts, in a process termed chlorophagy [49,50]. ATI1 also localizes to the ER and interacts with ER-localized ARGONAUTE1 (AGO1), suggesting a role as a selective cargo receptor for AGO1 [51]. Recently, Wu et al. demonstrated that ATI proteins participate in dark-induced ER-phagy, independent of ER stress [52]. Their results also suggested that this dark-induced ER-phagy requires the repression of TOR, a conserved kinase that negatively regulates autophagy in response to nutrients. The ER-tethered Membrane Steroid Binding Protein 1 (MSBP1) was identified as a new interactor of the ATI proteins, and autophagic turnover of MSBP1 depends on ATI proteins, suggesting a role for the ATI proteins in selective ER-phagy.

Thus, a number of different ER-phagy receptors have been found in both plants and other organisms, and there may be more yet to be identified. It appears that this diversity of receptors is needed for the degradation of different parts of the ER with different structural features, and also for the activation of ER-phagy under different stress conditions or in distinct cell and tissue types. For example, the localization of reticulons, and therefore their potential functions, is determined by their differential binding to membranes, depending on the membrane curvature. Reticulon proteins can also themselves lead to membrane curvature, and therefore may play a dual role in ER-phagy, both as receptors for incorporation of ER into autophagosomes and as factors that promote ER membrane remodeling and fragmentation [42,43]. In addition, when confronted with different types of biotic and abiotic stress, plants may activate ER-phagy via distinct regulatory mechanisms and using different receptors as a means of coordinating autophagy with other stress response pathways.

## 7. Conclusions and Future Perspectives

Plant ER-phagy has primarily been studied during ER stress, in which chemical agents, heat stress or pathogens lead to the accumulation of unfolded or misfolded proteins. Interestingly, in a recent study, dark treatment was found to trigger ER-phagy in a process dependent on the ATI proteins, but independent of ER stress [52]. In another case, local phosphate deficiency was able to activate ER-stress dependent autophagy. This process required a cell wall-targeted ferroxidase, Low Phosphate Response 1 (PLPR1), and an ER-resident P5-type ATPase Phosphate Deficiency Response 2 (PDR2), genes that function in phosphate deficiency rather than ER stress [53]. As a key ER stress sensor, IRE1b has been demonstrated to be required for ER stress-induced autophagy; the role of IRE1b in dark stress-induced ER-phagy is worth investigating. In general, how ER-phagy is activated in response to different environmental or cellular conditions, and its function in these conditions, remains unclear.

Beyond acting in stress responses, ER stress sensors are also critical for plant growth and development [54,55,56], but the underlying mechanism is still not well understood. Conventionally, the UPR is triggered by unfolded or misfolded protein accumulation caused by environmental stress [27]. In studying a triple mutant in which both arms of the UPR are blocked, we showed that an active UPR is also required for normal plant vegetative development, suggesting that there is a basal activity of the ER stress signaling pathways even without stress [55]. Basal autophagy activation has been demonstrated to be important for plant vegetative growth [57]; whether the basal activity of ER stress response components such as IRE1b can trigger ER-phagy during plant development is as yet unknown.

Two major pathways govern the degradation and recycling of unwanted components in eukaryotes, 26S proteasome-mediated degradation of target proteins after ubiquitylation [58], and autophagy-mediated turnover of bulk cargo including protein aggregates and damaged organelles [10]. Despite the broad conservation of the core autophagy machinery, many regulators of autophagy found in other organisms do not exist in plants [5]. Various types of selective autophagy appear to be present in plants, but we still lack a comprehensive understanding of these processes, and many of the relevant selective autophagy receptors are yet to be discovered. The best-studied plant selective autophagy receptor Neighbor of BRCA 1 (NBR1) directly interacts with ATG8 through its AIM motif, and also recognizes ubiquitylated protein aggregates, targeting them to the central vacuole for recycling [59,60,61]. Likewise, the proteasome subunit RPN10 functions as a bridge for selecting ubiquitylated 26S proteasomes for autophagy, and interacts physically with ATG8 through its UIM motif [14], suggesting these two pathways are highly coordinated. 

A number of receptors have now been proposed to function in selective autophagy of ER, as described above, but it is likely that the full complement of receptors is yet to be identified. Based on motif conservation and prediction, many other AIM- or UIM-containing proteins may exist in plants [13,14], whose functions in selective autophagy, including ER-phagy, remain to be verified. In addition, alternative motifs for interacting with ATG8 may exist. For example, the ER-phagy receptor C53 has an unusual AIM (a shuffled AIM), and we have recently identified a new ATG8-interacting protein Constitutively Stressed 1 (COST1) which has no obvious AIM or UIM motifs, raising the possibility that there might be additional interaction motifs that are distinct from AIM and UIMs [62,63]. Identification of new receptors and regulators of ER-phagy in plants will provide a better understanding of the mechanism and selectivity of this important process.
